# Mammalian–Environmental–DNA-Derived Probabilistic Modeling Reveals Re-Established Trophic and Co-Habitation Network Structure in Fire-Affected Terrestrial Communities

**DOI:** 10.3390/biology15201827

**Published:** 2026-10-13

**Authors:** Anh H. Nguyen, Kim Da Yun, Han Eunyoung, Seungdae Oh

**Affiliations:** 1 Department of Civil Engineering, College of Engineering, Kyung Hee University, Yongin-si 17104, Gyeonggi-do, Republic of Korea; 2 Hwajeong Engineering Co., Ltd., Hwaseong-si 18479, Gyeonggi-do, Republic of Korea

**Keywords:** eDNA, *COI*, MiMammal, Bayesian network, ecological health assessment

## Abstract

Climate change threatens natural ecosystems with more frequent and intense extreme weather events, including forest fires. This study evaluated mammalian communities, a primary bio-indicator of forest health, in Uljin Forest, using both traditional camera traps and advanced environmental DNA (eDNA) metabarcoding. Fire severity at the study sites was categorized based on field observations and satellite images using the differenced Normalized Burn Ratio. Water basins were installed for 7 days, after which the water was collected for eDNA extraction. A multi-primer approach using mitochondrial cytochrome c oxidase subunit 1 and *12S rRNA* genes was employed. Comparative analysis of the mammalian relative abundance indicated that while camera traps remain the optimal tool for monitoring large mammals, eDNA methods were significantly more effective for quantifying small, cryptic species. Although long-term effects remain observable in the biodiversity and abundance of mammalian communities, Bayesian network analysis at the order level inferred positive ecological signals three years post-fire, with the food-web-related and cohabitation dynamics re-established. eDNA metabarcoding improved mammal detection relative to traditional methods and enabled probabilistic modelling to reveal ecological structure that would not be possible solely with biodiversity analysis. Overall, this multi-primer/multi-analysis approach highlights the potential of eDNA for ecosystem health monitoring.

## Simple Summary

Under the effects of climate change, monitoring methods for bioindicators of ecosystem integrity have become essential to support conservation initiatives and mitigate consequences such as habitat loss or the emergence of invasive species. This study investigates the use of both traditional (camera traps) and advanced environmental DNA (eDNA) metabarcoding methods with a multi-primer approach to evaluate the mammal communities of different forest fire severities. The results showed that the traditional methods were effective in monitoring large mammals, while small and cryptic species were better monitored with the eDNA methods. Moreover, eDNA methods enabled the assessment of the complex relationships within eukaryotic communities, inferring positive ecosystem signals 3 years post-fire. The plants’ regrowth was affected by the early stages of the post-fire recovery process, while the food-web-related or habitat–occupation interactions of other eukaryotes were re-established regardless of the fire severity. These findings highlight the potential of eDNA for ecosystem health monitoring under climate change’s impacts, with invaluable insights into the complex eukaryotic relationships that would be difficult to assess using traditional methods.

## 1. Introduction

Climate change affects both scientific and global socioeconomic frameworks, particularly due to its impact on the frequency and magnitude of extreme weather events [1]. The direct ecological consequences of these events include a loss of habitat, the redistribution of organisms, and the emergence of niche or invasive species [2]. To mitigate these impacts and support conservation initiatives, the long-term monitoring of ecosystem integrity has become essential. A critical bio-indicator of this integrity is the biodiversity of forest eukaryotes, including mammalian communities. Conventional mammalian monitoring approaches are typically based on the use of camera traps and voice recorders to evaluate mammalian distribution, behavior, and diversity [3]. However, the detection probability of these methods can vary by species, method, or observer skill, while environmental factors such as the weather, light, and/or terrain can affect data quality and/or equipment performance. Traditional surveys are also prone to false negatives, with particular difficulty documenting cryptic, low-density, and/or silent species [4]. Due to these limitations, environmental DNA (eDNA) has emerged in recent years as a complementary monitoring method. By analyzing genetic material collected from environmental matrices (e.g., skin, scales, and excrement), eDNA represents a non-invasive and cost-efficient method for assessing population genetics and community composition. eDNA is particularly useful for the detection of transient species that are not physically present during sampling. When coupled with the development of taxa-specific primers, this approach significantly enhances detection sensitivity for rare species that are often overlooked by traditional surveys [5].

Despite the considerable potential of eDNA-based monitoring, significant research gaps exist, particularly for terrestrial vertebrates. Terrestrial mammals typically deposit lower concentrations of genetic material into aquatic matrices, which are a common sampling medium for environmental surveys. Thus, while various studies have extensively characterized the diversity and structure of aquatic communities in high resolution using this approach [6,7], current research on terrestrial mammals is often limited to occupancy modeling and frequently lacks comprehensive data on community composition and overall biodiversity [8,9,10,11,12,13]. As a result, aquatic organisms such as cyanobacteria, algae, fungi, fish, and aquatic mammals (e.g., dolphins and whales) have been analyzed in detail, including their relative abundance at different taxonomic levels [14], ordination analysis (e.g., NMDS and PCoA) [15,16], alpha diversity [17], and interactions among detected organisms. However, the listed interactions among the aquatic organisms have generally been limited to linear correlations or food-web dynamics, often overlooking the impacts of stochastic environmental disturbances. Of the few studies that have addressed terrestrial mammalian interactions, some have successfully utilized multi-scale diversity metrics (e.g., alpha, beta, and gamma diversity) and ordination techniques. However, these remain centered on trophic networks (i.e., the diets of carnivores and omnivores) [18] or patterns of co-occurrence among mammals [19], thus failing to account for the pressures exerted by natural disasters. Furthermore, while bioinformatics artifacts are a persistent concern in eDNA methodology, they remain under-addressed in existing literature. Various primers have been utilized in different studies, but their specific taxonomic resolution has rarely been reported, limiting their utility as benchmarks for future research. As a collective result of these methodological limitations, a holistic understanding of the community composition and ecosystem interactions of terrestrial mammals under the stressors of climate change and more intense natural disasters remains unexplored.

Given this context, the present study aims to enhance the understanding of how forest fires impact the community composition, biodiversity, and behavior of terrestrial mammals using a multi-method approach, employing both camera traps and eDNA. To assess this, water basins were deployed along with camera traps at various sites in Uljin Forest that were stratified by the severity of fire damage three years after a forest fire. Following a 7-day exposure period, aqueous samples were collected from the basins for eDNA extraction and subsequent high-throughput sequencing. To maximize detection rates and ensure comprehensive community profiling, a multi-primer strategy was employed, utilizing both a universal primer and a mammal-specific primer set. Probabilistic network modeling was also used to construct causal relationships between fire intensity and established ecological interactions among a diverse group of terrestrial taxa, including the mammal, floral, and insect communities. In addition, by constructing phylogenetic trees, genuine biological sequences in the eDNA data were distinguished from potential artifacts, thus confirming the taxonomic findings and the robustness of the eDNA method. This integrative approach aimed to assess the functional recovery of the ecosystem three years post-fire. This form of analysis is required to quantify ecosystem resilience and recovery trajectories following a forest fire, thus providing novel insights into the complex interspecific interactions that define terrestrial forest biomes.

## 2. Materials and Methods

### 2.1. Study Area, Basin Installation, and Camera Trapping

**Figure 1:**
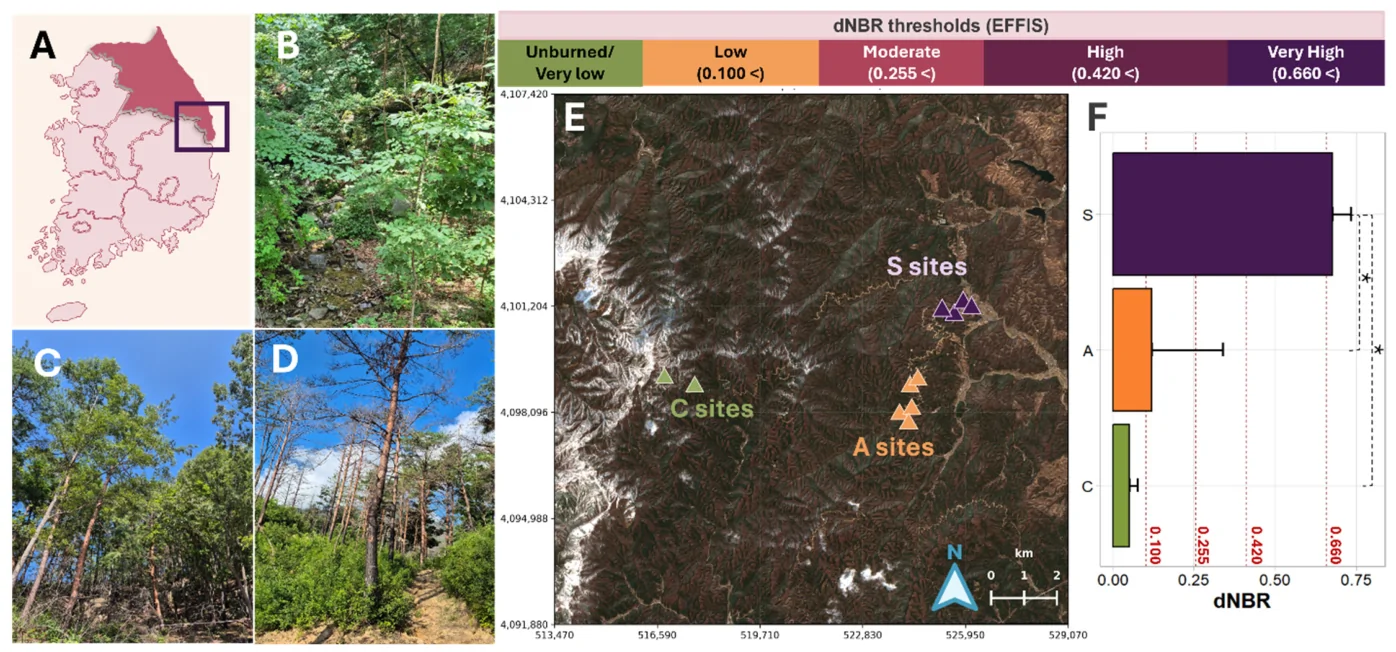
Study area and fire severity classification. (**A**) Location of the study area on the Korean Peninsula. Gangwon Province is colored pink, and the location of the Uljin forest area in Gangwon Province is highlighted in the purple square. Field observations at (**B**) control, (**C**) affected, and (**D**) severely burnt sites. (**E**) Spatial distribution of the sampling sites within the study area. The areas of the sites were marked as C (control), A (affected), and S (severely burnt) sites. (**F**) Averaged differenced Normalized Burnt Ratio (dNBR) at the C, A, and S sites collected from the Sentinel-2 database. The bar plots were colored according to fire severity, as determined using the dNBR thresholds established by the European Forest Fire Information System (EFFIS): C in green, A in orange, and S in purple. The bars and error bars represent the mean and ±1 standard deviation from the mean. An asterisk denotes a statistically significant difference (*p* < 0.05).

Study sites were selected in Uljin Forest, Gangwon Province, Republic of Korea (Figure 1A). This is a mixed forest with 54.9% of the forested area consisting of broadleaf trees [20]. A wildfire that occurred in 2022 reportedly damaged approximately 17,000 hectares of this forest, severely interfering with the native ecosystem [21]. Field surveys were used to select 11 sites, representing different levels of fire severity. The sites were later classified as control (C), affected (A), and severely burnt (S) areas, with a distance between the C sites and the A and S sites of 7–8 km, while the distance between the A and S sites was approximately 2.5 km. Initially, 24 water basins (2.5 L) were installed (i.e., 8 at C sites, 8 at A sites, and 8 at S sites) with the rim at ground level on known mammal trails that lack natural water sources to increase their exposure to mammals. After 7 days, due to basin loss, we collected water from 2 basins at C, 5 basins at A, and 4 basins at S for eDNA harvesting and subsequent analysis. Basins with compromised or insufficient water volume for further analysis were excluded to ensure sample integrity and reliability of eDNA detection. The total of 13 basins lost across all sites were likely due to animal spillage and evaporation during the deployment period. However, the basins were only checked at the collection time, and the two mechanisms leave similar evidence (i.e., empty or partially empty basins), the specific cause for each basin was not assigned.

Camera traps were deployed for 1 month at 11 sites to capture images of mammals. Each site was set up with three camera stations, and the total trap-nights were 30 nights. The camera model used was the Bushwhacker Big Eye D3 (Shenzhen Welltar Electronic Technology Co., Ltd., Shenzhen, China), which features infrared LEDs for nighttime image capture. In Camera mode, the camera captured 1 photo per trigger (30-s trigger interval) at normal sensitivity [9,10]. The picture quality was set at 12 MP (3024 × 4032). Color pictures were taken during the day, while monochrome pictures were taken at night. The cameras were mounted 0.5–1.1 m above ground, angled to face possible mammal trails where animals were likely to be encountered [22]. No bait or lures were used. Manual examination was conducted to identify the mammals to the highest taxonomic level based on their morphology. Independent detection intervals for large mammals (e.g., goat, deer, badgers) were 60 min, and for small mammals (e.g., squirrels) were 15 min [23]. The relative abundance of the camera-detected mammals was calculated as a proportion of the total detections.

### 2.2. Study Sites Classification with Satellite Imagery Assessment

The satellite images from the Sentinel-2 mission were employed for the quantification of the fire-severity levels. Level-A2 surface reflectance imagery was corrected for atmospheric, geometric, and radiometric distortions. The specific fire event and the sensing dates are listed in Table 1.

In brief, pre-fire imagery was acquired one year prior to the event, while the post-fire period was at the sampling period, three years after the fire, with a one-month sensing window. The differenced Normalized Burnt Ratio (dNBR) was calculated using the following equations to evaluate the fire severity [24]:

(1) $$\mathrm{NBR}=\;\frac{\mathrm{NIR}-\mathrm{SWIR}}{\mathrm{NIR}+\mathrm{SWIR}}$$

*dNBR* = *NBR_pre_*_−*fire*_ − *NBR_post_*_−*fire*_ (2)

where *NIR*, *SWIR*, and *RED* are the Sentinel-2 bands with their respective central wavelengths and spatial resolution (Table 2). *NBR_pre-fire_* and *NBR_post-fire_* were derived from the pre-fire and post-fire imagery, respectively. Fire-severity classification was based on thresholds established by the European Forest Fire Information Service (EFFIS) (Figure 1F) [25]. Mann–Whitney U tests (*p*-value threshold = 0.05) were used to identify statistical differences between the experimental conditions.

### 2.3. Bioinformatics Analysis

The basin water samples were collected, transported, and stored at 4 °C in the laboratory until processed (<48 h). The eDNA was captured using 0.22-μm cellulose nitrate membrane filters, and a SPINeasy DNA Kit for Water (MP Biomedicals, Seoul, Republic of Korea) was employed for eDNA extraction following the manufacturer’s protocols [26]. A NanoDrop spectrometer (Biolab Scientific Ltd., Scarborough, ON, Canada) was used to quantify the concentration and purity of the extracted DNA, which was subsequently PCR-amplified for the mitochondrial gene cytochrome c oxidase subunit 1 (*COI*) and mitochondrial *12S rRNA* using the coupled COI-C01 (5′-TYTCWACWAAYCAYAAAGAYATTGG-3′) and COI-C03 (5′-ACYTCYGGRTGACCAAARAAYCA-3′) primers and MiMammal (forward: 5′-ACACTCTTTCCCTACACGACGCTCTTCCGATCTNNNNNNGGGTTGGTAAATTTCGTGCCAGC-3′; reverse: 5′- GTGACTGGAGTTCAGACGTGTGCTCTTCCGATCTNNNNNNCATAGTGGGGTATCTAATCCCAGTTTG-3′) primer, respectively [27,28], and run through the Miseq sequencing platform (Illumina, San Diago, CA, USA). The amplification mixture contained 10 ng gDNA and 5× reaction buffer (1 mM dNTP mix, 500 nM each of the universal F/R PCR primers, and Herculase II fusion DNA polymerase (Agilent Technologies, Santa Clara, CA, USA)). The PCR mixture was denatured at 95 °C for 3 min, followed by 25 cycles of 30 s at 95 °C, 30 s at 45 °C (*COI*), 65 °C (MiMammal), and 30 s at 72 °C, followed by a 5-min final extension at 72 °C. All PCR reactions were triplicated to ensure consistency. The quantity and quality of the final products were checked using the qPCR Quantification Protocol Guide (KAPA Library Quantification kits for Illumina Sequencing platforms, Roche Diagnostics Korea Co., Ltd., Seoul, Republic of Korea) and TapeStation D1000 ScreenTape (Agilent Technologies, Waldbronn, Germany), respectively. Negative controls (n = 2) for each step of sample collection, filtration, extraction, and PCR, with deionized water replacing the actual samples, were handled using the same protocol as the actual samples to assess possible contamination [29,30].

The raw *COI* and MiMammal sequence data were also analyzed using the Pipeline for Environmental DNA Metabarcoding Analysis (PEMA) with default parameters [31]. In brief, reads were quality-checked (FASTQC 0.11.9) and trimmed (Trimmomatic 0.39) to remove low-quality bases and ambiguous reads. The naiveBayes algorithm, PANDAseq, and OBItools were subsequently used to denoise, merge, and dereplicate the retained sequences using the naiveBayes algorithm, respectively. Taxonomic assignments of the eukaryotic sequences in the NCBI nr/nt database were carried out with BLASTn 2.16.0 [32]. The identity cut-off at the order, family, genus, and species level was 85%, 90%, 95%, and 98% for *COI* and 85%, 90%, 97%, and 99% for MiMammal, respectively, in accordance with previous studies [33,34]. Relative abundance of each mammalian order was normalized by the total read counts of all mammalian orders at each fire severity category for each monitoring method. Diversity analyses (i.e., NMDS and alpha diversity) were conducted with read counts data at the order level using a random sampling method with z-score transformation and Gaussian jittering by 0.3 of one standard deviation for 10 times, then transformed back to its original scale.

### 2.4. Bayesian Network Probabilistic Modelling

Bayesian network was conducted to analyze the restoration dynamics among the eukaryotic organisms at the study sites and dNBR effects on the organisms three years post-fire by outputting a directed acyclic graph (DAG). The relative abundance of the targets (i.e., mammals and their food-chain-related counterparts) was used as input data for Bayesian network modelling using the bnlearn package in R software (version 4.6.1) [35]. dNBR was set as the root, while the eukaryotic organisms were listed in tiers according to their ecosystem functioning roles (i.e., primary producers, primary consumers, and secondary consumers) to avoid violating known ecological theories. The directionality of the nodes was not specified to allow the unbiased exploration of the complex multidirectional interactions between the eukaryotic organisms. Network construction was done with the tabu search (TS) algorithm with the Bayesian information criterion (BIC) as the scoring parameter to generate the DAG, presenting a posterior probability function while preventing overfitting [36]. Using the TS algorithm, initial DAGs were constructed, and the networks (e.g., nodes and edges) were optimized to reach the highest possible score. The bootstrapping method (1000 times) was carried out to construct the final reliable DAG, with boot.strength function identifying the strong edges (threshold = 0.6). The averaged.network function was employed to create the final Bayesian network model consisting of arcs present in more than half of the bootstrap networks (strength > 0.5). Model stability was assessed by repeating the full bootstrap 10 times with different random seeds and by counting retained arcs at strength thresholds 0.30 to 0.95 (Table S2). Sensitivity analyses reran the bootstrap with one element changed at a time: removing the blacklist, replacing AIC-g with BIC-g, replacing TS with hill-climbing, and applying a Centered Log Ratio (CLR) transformation to taxon abundances (pseudocount = half the smallest non-zero values; dNBR not transformed) [37]. Each network is combined with the baseline (Table S3a,b).

Pearson’s r and Spearman’s *p* between connected nodes were calculated as descriptive measures of the direction and magnitude of associations, with 95% percentile bootstrap confidence intervals (CIs). Tests of zero correlation were performed for all 91 node pairs and adjusted with the Benjamini–Hochberg (BH) procedure [38]. Since the arcs were selected from the same data, the *p*-values are descriptive. To assess whether arc strength exceeded those expected by chance across all candidate connections, each variable was permuted independently (50 permutations), the full bootstrap procedure including the blacklist was repeated on each permuted dataset, and the maximum arc strength was recorded (Table S1). The family-wise permutation *p*-value of an arc is (1 + number of permutations with a maximum strength at least as large as the arc’s)/(1 + 50) [39].

## 3. Results

### 3.1. Quantification of Long-Term Wildfire Perturbation on Forest Vegetation

Vegetation and mammal distributions in a forest ecosystem are strongly influenced by direct producer–consumer dynamics [40]. Thus, the observation and classification of forest fires based on the vegetation are essential for mammal-centered studies. Field assessment of the vegetation in Uljin Forest (Figure 1A) was thus carried out during the study period (i.e., three years post-fire). The C sites (Figure 1B) were characterized by intact herbaceous, shrub, and tall woody tree layers. At the A sites, damage from the fire was observed in the lower vegetation, with high leaf layers remaining on the tall woody trees but a significant absence of herbaceous plants or shrubs (Figure 1C). In contrast, the lower vegetation at the S sites was thriving, though many bare patches were observable and the majority of the tall woody trees remained lifeless (Figure 1D). These observations were also reflected in satellite image analysis. The approximate location of the sites was used to calculate the dNBR values (Figure 1E,F and Figure S1D), which were determined to be 0.051 ± 0.026 for the C sites, 0.120 ± 0.219 for the A sites, and 0.679 ± 0.055 for the S sites. Using the thresholds established by the EFFIS [25], the C sites were classified as Unburned/Very Low, while the A and S sites were classified as Low and Very High, respectively. Of note, the dNBR maps show variable values across A sites (Figure S1B), whereas the C (Figure S1A) and S (Figure S1C) sites appear more uniform. Figure S1E,F further elucidate the heterogeneous fire effects at the A sites: sites with low dNBR values had dense, healthy vegetation, while the site with high dNBR had less healthy vegetation, and no-canopy patches caused by dead trees remained. The combination of on-site observations and satellite images completed the site classification by fire severity.

### 3.2. Long-Term Effects of Forest Fire on Mammalian Biodiversity

The mammalian community structure across the study sites was characterized using a comparative framework of traditional and eDNA-based methodologies. None of the negative controls showed amplification of the targeted organisms’ DNA. The high-throughput sequencing generated 2,076,377 raw reads for *COI* samples, with a sequencing depth of 46–82 Mbps, and obtained 45,825 eukaryotic ASVs after bioinformatic analysis. MiMammal yielded 2,062,761 raw reads with a sequencing depth of 25–112 Mbps. 344,082 eukaryotic ASVs were obtained after bioinformatic analysis.

**Figure 2:**
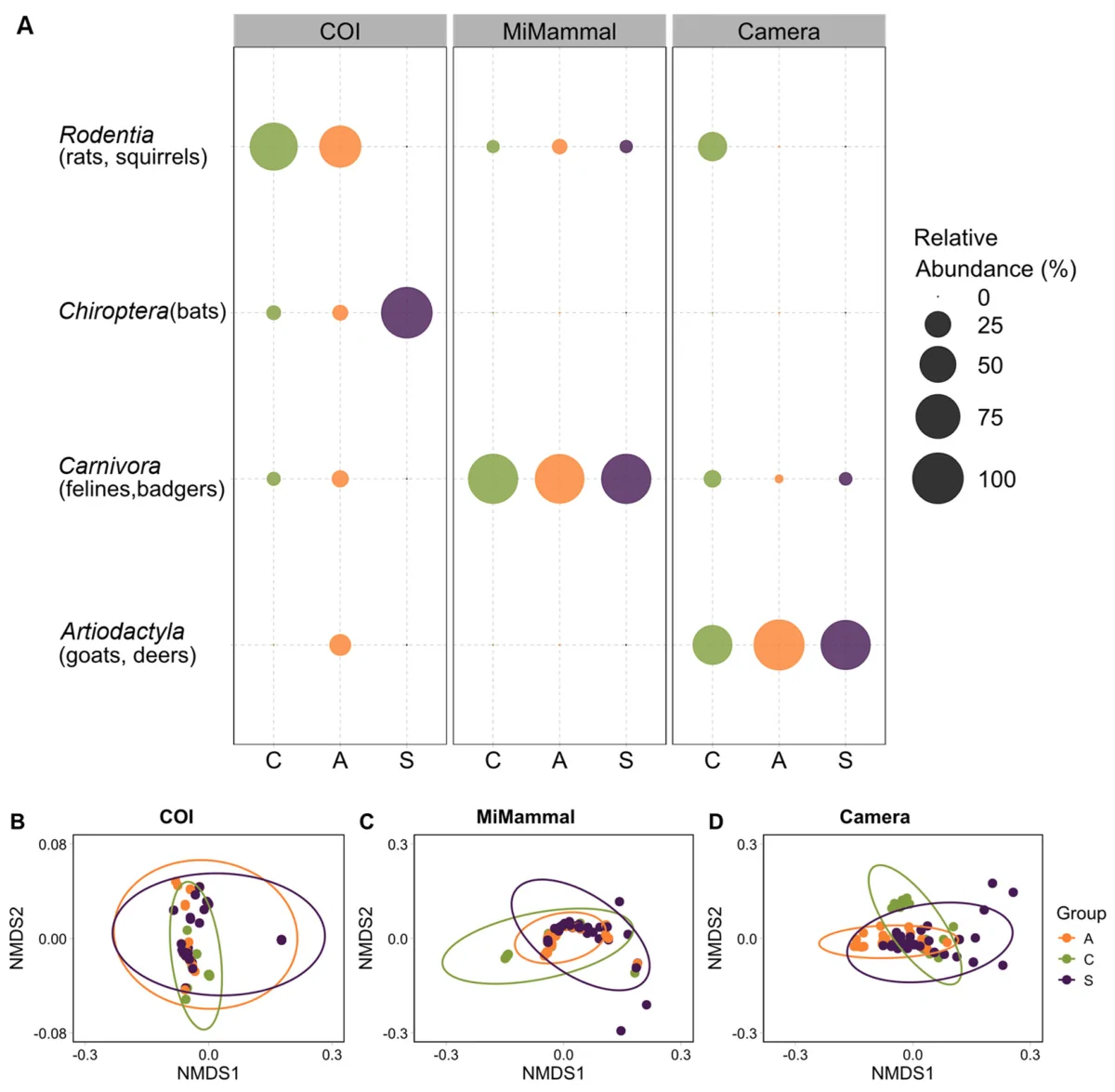
Mammalian community characteristics according to the assessment method. (**A**) Relative abundance of mammalian orders identified using *COI* metabarcoding, MiMammal metabarcoding, and camera trapping. NMDS plot using the Bray-Curtis distance for (**B**) *COI* eDNA, (**C**) MiMammal eDNA, and (**D**) camera traps.

Figure 2A presents the relative abundance of mammals at the order level. Four orders (Rodentia, Chiroptera, Carnivora, and Artiodactyla) were captured by the *COI* primer, with all four found at the A sites, 3 (Rodentia, Chiroptera, and Carnivora) at the C sites, and 1 (Chiroptera) at the S sites—making A sites the sites with the highest number of orders detected. Rodentia has the highest relative abundance at the C and A sites (86.67% and 65.86%, respectively). MiMammal captured only two orders (Rodentia and Carnivora) for all sites, with the Carnivora abundance (92.34–94.64%) considerably higher than that of Rodentia (5.352–7.651%). Compared to the molecular methods, the visual survey camera trapping documented Carnivora and Artiodactyla at all three site types, while Rodentia was only sighted at C sites; thus, C is the site that observed the highest number of orders. Artiodactyla had relative abundances of 97.91% and 94.23% at A and S sites, respectively. Despite these differences, ordination-based NMDS clustering analysis produced similar results between the two primers and between the molecular and the camera methods (Figure 2B–D). Though the MiMammal and camera methods separated C and S from the main cluster, the 95% confidence interval ellipses for all methods overlapped considerably, indicating little to no difference in beta diversity between the sites. Overall, *COI*-based monitoring produced the highest coverage of the detected orders (4) across all sites and was most sensitive to traces of mammals in the environment.

**Figure 3:**
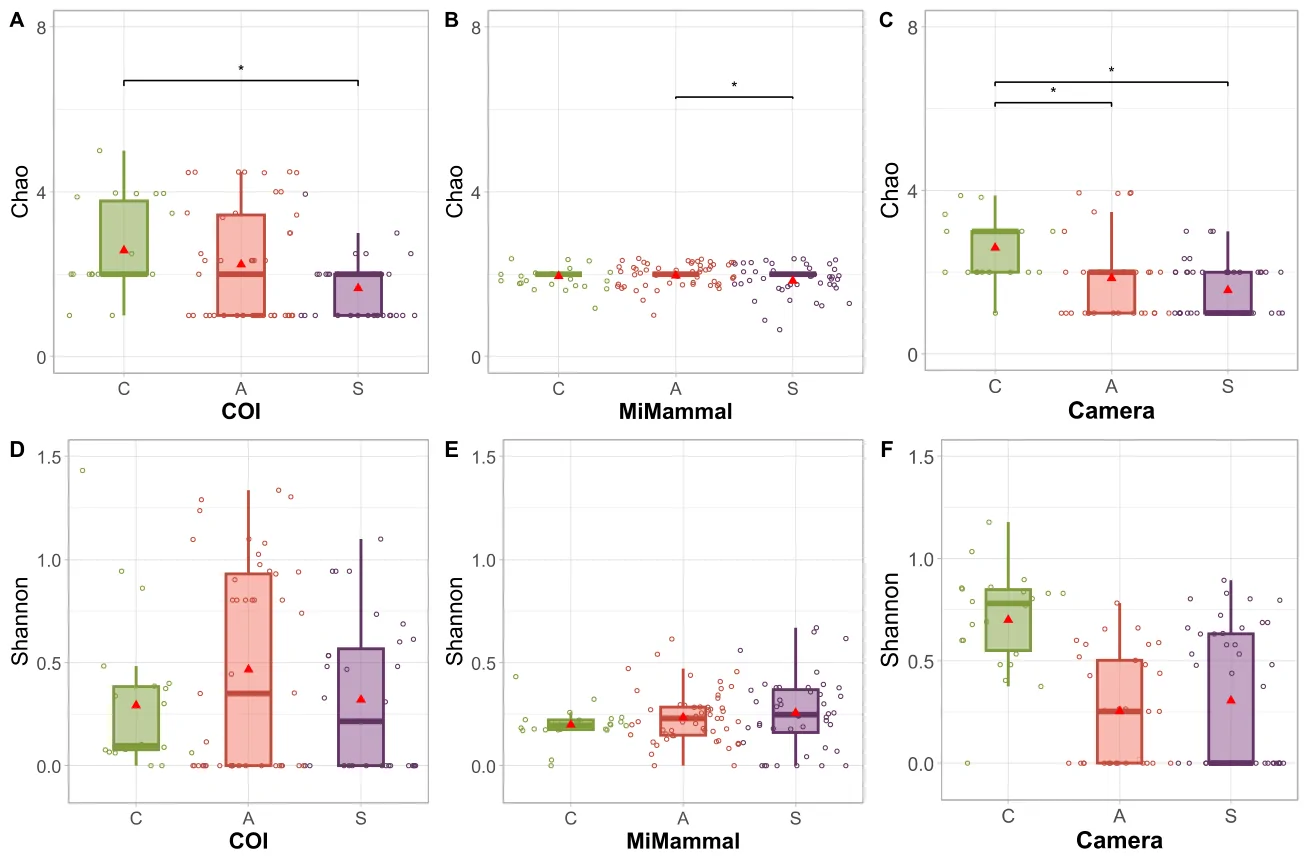
Alpha diversity indices of the mammalian community according to the assessment method. (**A**–**C**) Chao richness index. (**D**–**F**) Shannon diversity index. An asterisk denotes a statistically significant difference (*p* < 0.05), and the mean is presented as a red triangle.

**Figure A1:**
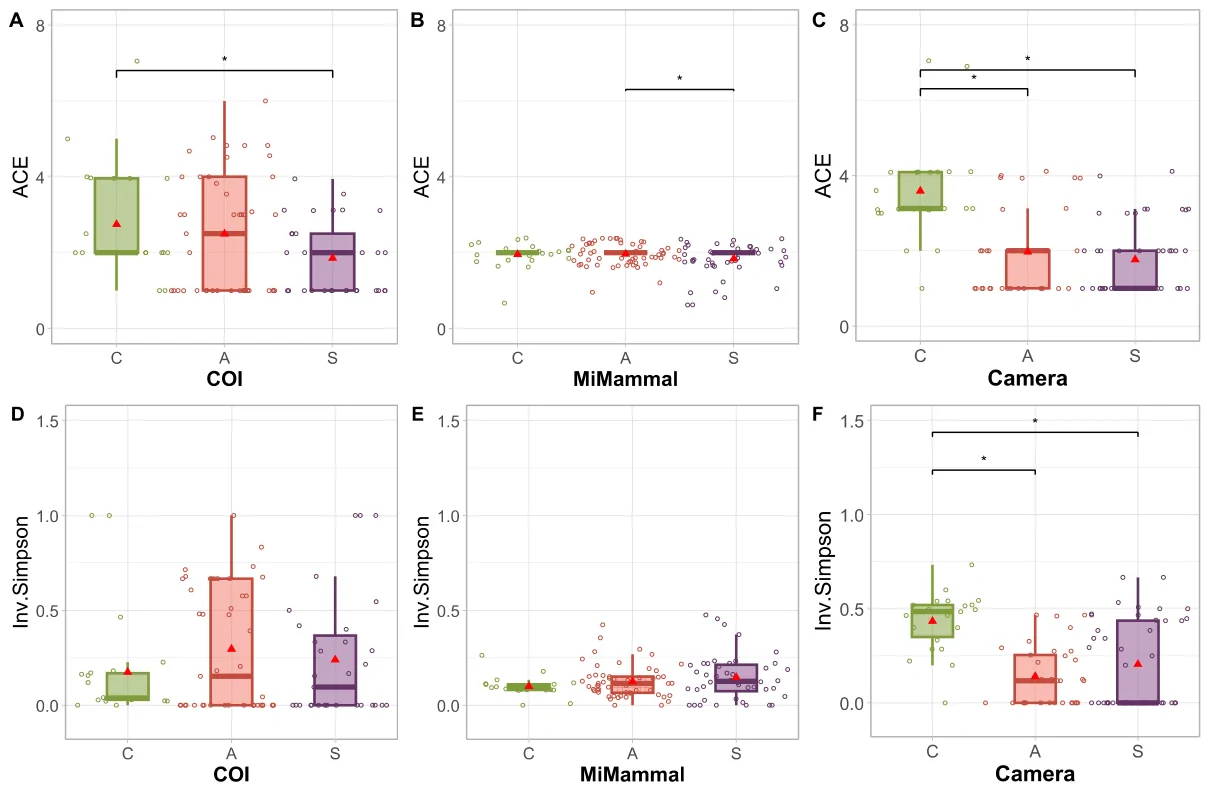
Alpha diversity indices of the mammalian community according to the assessment method. (**A**–**C**) ACE richness index. (**D**–**F**) Inv. Simpson diversity index. An asterisk denotes a statistically significant difference (*p* < 0.05), and the mean is presented as a red triangle.

Alpha diversity analysis was conducted to assess the species diversity and richness in the study area. Comparing the two molecular methods, Chao (Figure 3A) and ACE (Figure A1(A)) richness indices revealed a clear decrease by fire severity level in the *COI* data, with a statistically significant difference between the C and S sites for both indices, whereas the A sites exhibited considerable fluctuations. MiMammal (Figure 3B and Figure A1(B)) yielded relatively uniform richness values between sites (approximately 1.8–1.9), though the differences between the A and S sites were statistically significant (*p* < 0.05). Conversely, the camera data (Figure 3C and Figure A1(C)) demonstrated a sharp reduction in richness as fire severity increased, with statistically significant differences observed for C vs. A and C vs. S, while the difference in richness between the A and S sites was low (1.855 ± 1.007 and 1.561 ± 0.575 for Chao, and 1.972 ± 1.130 and 1.76 ± 0.915 for ACE, respectively) with no statistical significance. The trends observed for the diversity indices Shannon and inverse Simpson were similar to those for Chao and ACE in the metabarcoding methods. The *COI* data (Figure 3D and Figure A1(D)) exhibited strong fluctuations at the fire-damaged sites, while the C sites had high diversity levels. MiMammal produced similar results, although the absolute diversity was highly consistent across sites (Figure 3E and Figure A1(E)). However, the camera trap data (Figure 3F and Figure A1(F)) revealed a clear reduction in diversity due to fire disturbance. This was most evident in the inverse Simpson index, which fell from 0.433 ± 0.159 at the C sites to 0.141 ± 0.159 and 0.205 ± 0.231 at the A and S sites, respectively. Collectively, these results indicate that the *COI* primer captured the higher within-sample diversity, confirming its superior sensitivity for detecting fine-scale community-level variation compared to MiMammal.

### 3.3. eDNA and Network Analysis of the Interactions Among Forest Eukaryotes

**Figure 4:**
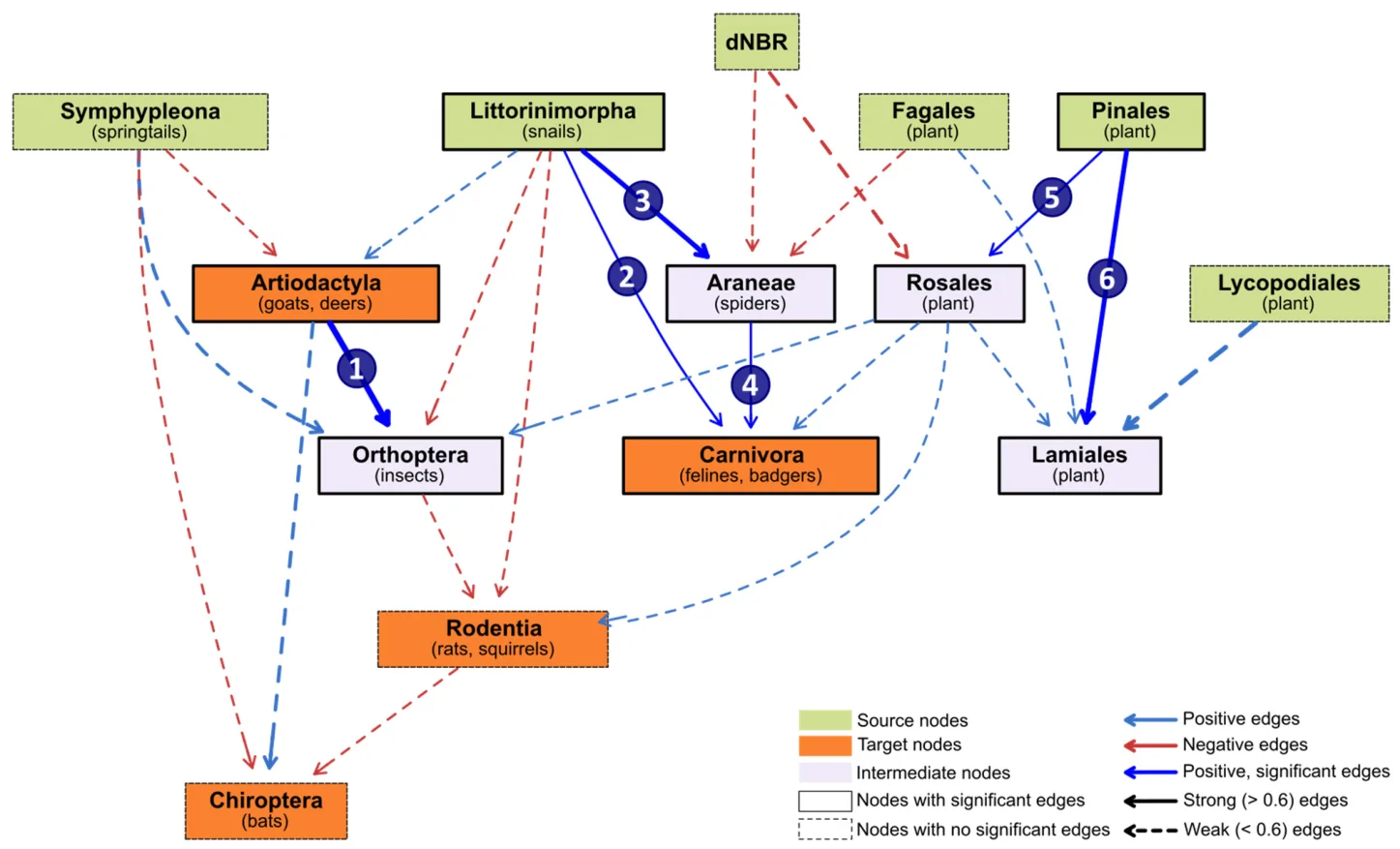
Bayesian network illustrating the associations among eukaryotic orders. The statistical significance of the edges was tested using Pearson’s r values. Edges representing statistically significant associations are labeled with numerical identifiers.

eDNA metabarcoding using the universal *COI* primers (i.e., COI-C01 and COI-C03) allows the capture of other non-mammal DNA signals and investigates the ecosystem relationships between mammals and other living organisms at the order level within the forest sites based on the Bayesian network method (Figure 4). Nodes were selected based on food-chain classifications, targeting producer–consumer and predator–prey dynamics. dNBR values were also included as environmental factors directly reflecting fire severity. The network was robust to the scoring function and to the search algorithm, but less so to the removal of the blacklist and to CLR transformation (Table S3a,b). The permutation test resulted in an arc strength median of 0.86 and a 95th percentile of 0.94, ranging from 0.75 to 0.98, which is comparable to the strongest observed arc of 0.88 (Table S1). Using Pearson’s R > 0.6 as the threshold for statistical significance, six linkages were found to be significant within the constructed network, and the arcs’ strengths were reproducible across 10 repeated runs (100% of runs). Of these, linkages 1, 5, and 6 were classified as species–habitat associations (i.e., relationships between habitat features and the occurrence of the target species [41]), while linkages 2, 3, and 4 were classified as predation–prey associations (i.e., one free-living organism kills and derives resources from another organism [42]). Positive correlations were observed between Artiodactyla and Orthoptera and between Pinales and both Rosales and Lamiales (arc strengths 0.88, 0.76, and 0.68, respectively). Littorinimorpha and Aranea also exhibited a positive association with each other and with Carnivora (arc strengths 0.61, 0.59, and 0.81, respectively). While Pearson’s adjusted *p*-values of linkages 2, 3, 4, and 5 were <0.05, Spearman’s adjusted *p*-values for all arcs were >0.05, and the permutation family-wise *p*-values were 0.37–1.00. Thus, the network was utilized for exploratory analysis of the current eukaryotic interactions. Of note is that dNBR had no direct linkage to any nodes, and no direct connections were found between plant orders and Artiodactyla. These results suggest that, during the study period, any signals of fire severity interfering with ecological interactions were negligible. Therefore, while the change in diversity and richness due to the fire event remained, it appears as if the ecosystem functions had been mostly re-established three years after the fire.

### 3.4. Taxonomic Resolution of the Monitoring Methods

**Figure 5:**
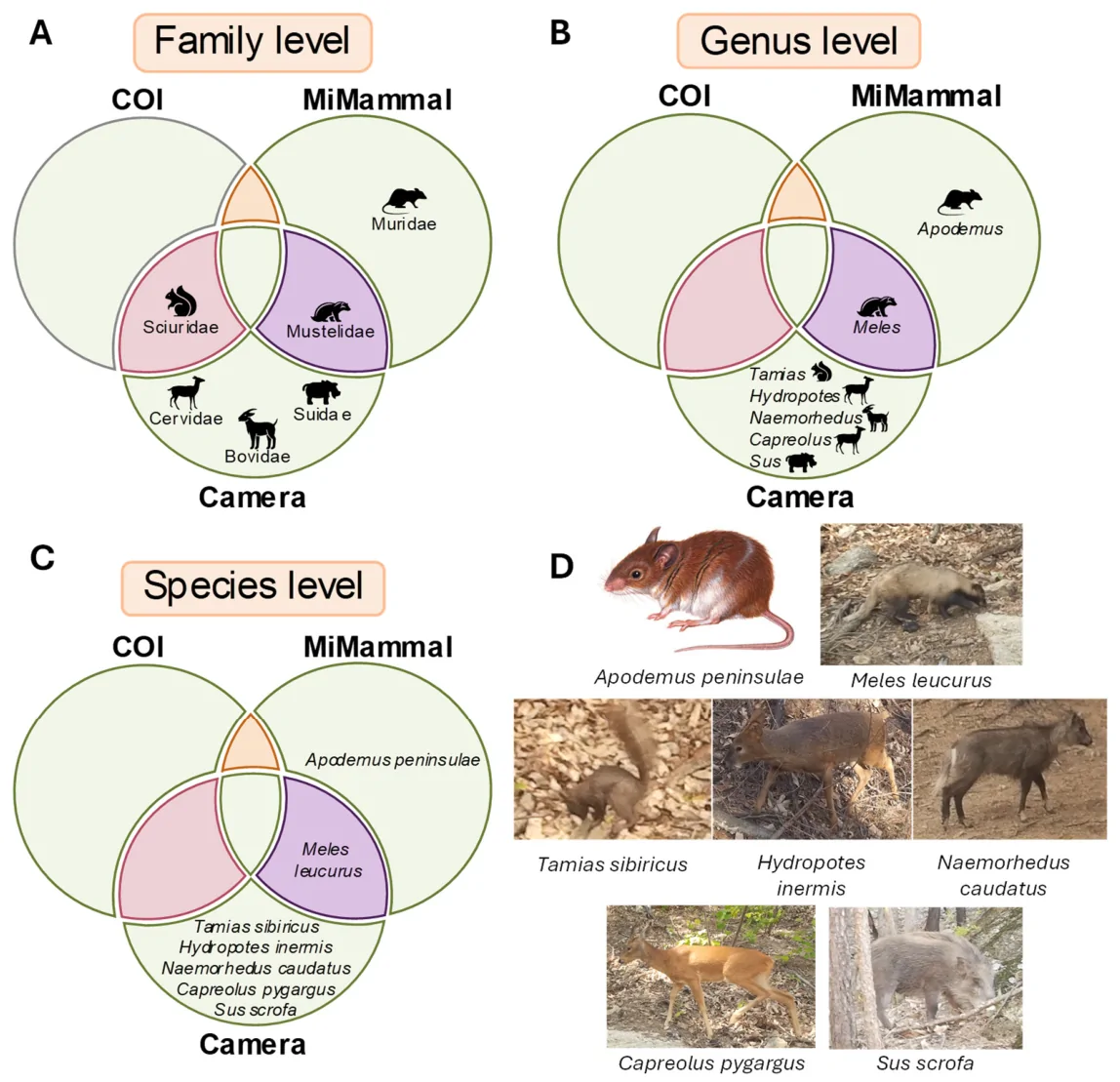
Venn diagram of mammalian taxa detected using traditional camera traps and eDNA-based methods at different resolutions. (**A**) Family level. (**B**) Genus level. (**C**) Species level. (**D**) Actual pictures of mammals captured using camera traps during the study period. Morphological description of *Apodemus peninsulae* was taken from Mammals of Korea [43].

The taxonomic resolution of eDNA-based monitoring remains a primary concern for its widespread application. Figure 5A–C illustrates the identification resolution of the three methods used in this study at the family, genus, and species levels. At the family level, both *COI* and MiMammal metabarcoding identified two families that were also captured using the camera method (Scriuridae and Mustelidae, respectively). MiMammal identified an additional family (Muridae), while the camera recorded three additional families (Cervidae, Bovidae, and Suidae) that were not detected using the other methods. At the genus level, no *COI* sequences met the requirements for identification, while MiMammal identified two genera (*Meles* and *Apodemus*) and the camera traps identified five genera (*Meles*, *Tamias*, *Hydropotes*, *Naemorhedus*, *Capreolus*, and *Sus*). At the species level, *Apodemus peninsulae* and *Meles leucurus* were identified using MiMammal, while *M. leucurus*, *Tamias sibiricus*, *Hydropotes inermis*, *Naemorhedus caudatus*, *Capreolus pygargus*, and *Sus scrofa* were identified using the camera traps. Overall, *M. leucurus* was detected at a high resolution and was the only species that was detected using both the MiMammal and camera methods. Figure 5D presents an example of *A. peninsulae* [43], while actual images of the other species captured at the study sites are shown.

**Figure 6:**
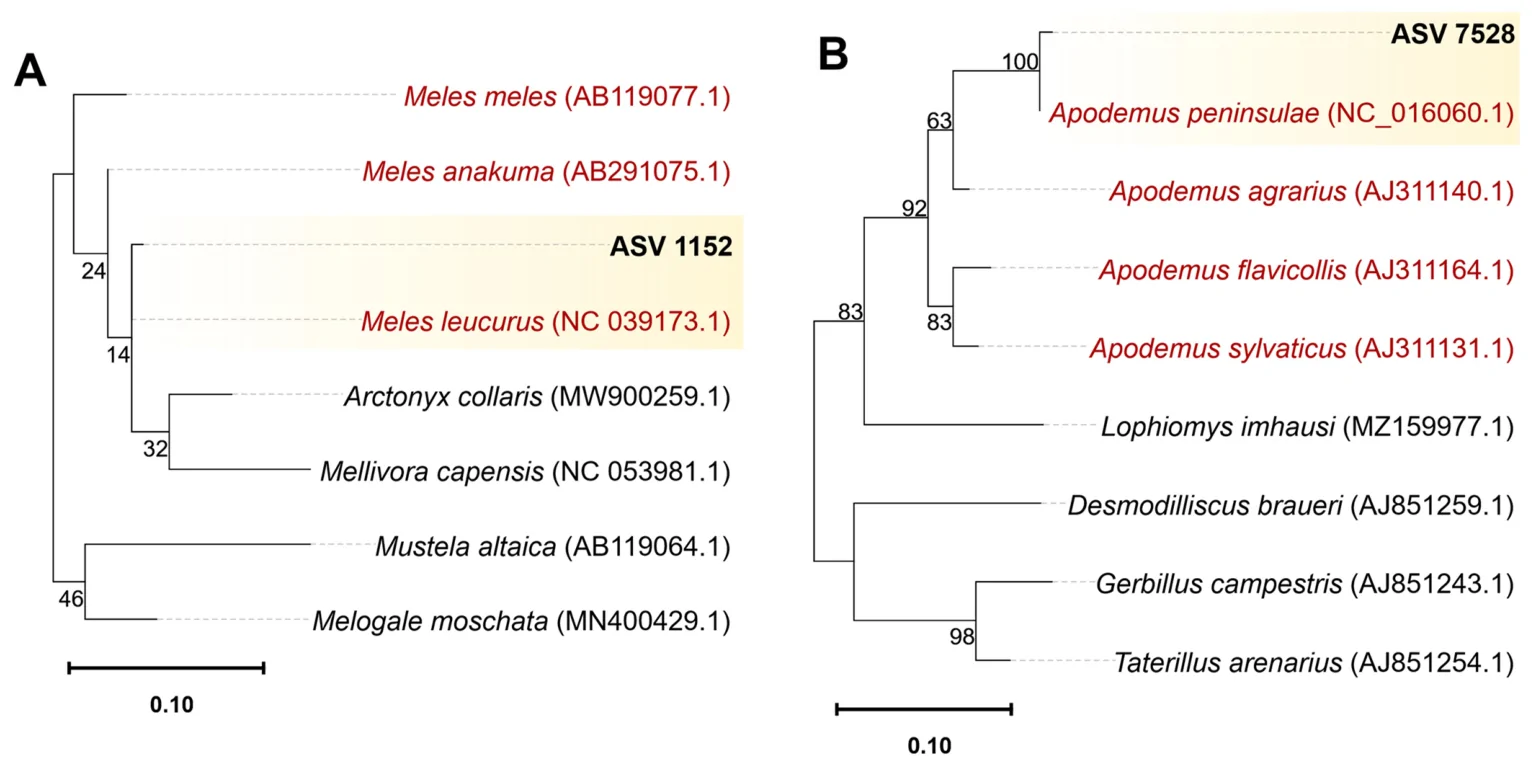
Phylogenetic tree of the MiMammal-identified ASVs and reference sequences within the families Mustelidae and Muridae. (**A**) ASV 1152 and Mustelidae reference sequences. (**B**) ASV 7528 and Muridae reference sequences. The phylogenetic tree was constructed using the 12S mitochondrial rRNA gene amplified with the MiMammal primer. Reference sequences with the accession number in parentheses were collected from a non-redundant nucleotide database (nr/nt). Phylogenetic reconstruction was conducted using the maximum likelihood method under the Tamura–Nei substitution model in MEGA12. Sequences from the same genus are highlighted in red. The colored box shows sequences originating from Korea.

**Figure A2:**
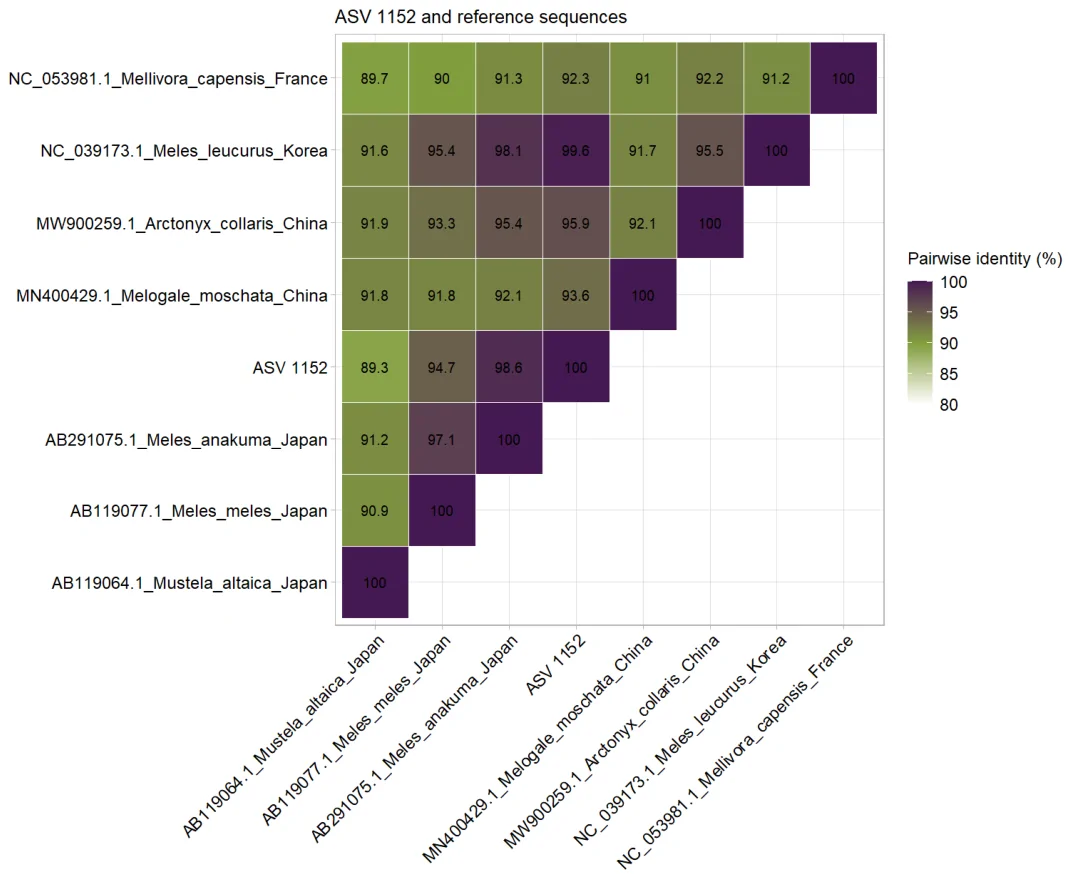
Pairwise sequence identity of ASV 1152 with reference sequences from the family Mustelidae. The colour key indicates the pairwise percent identity.

**Figure A3:**
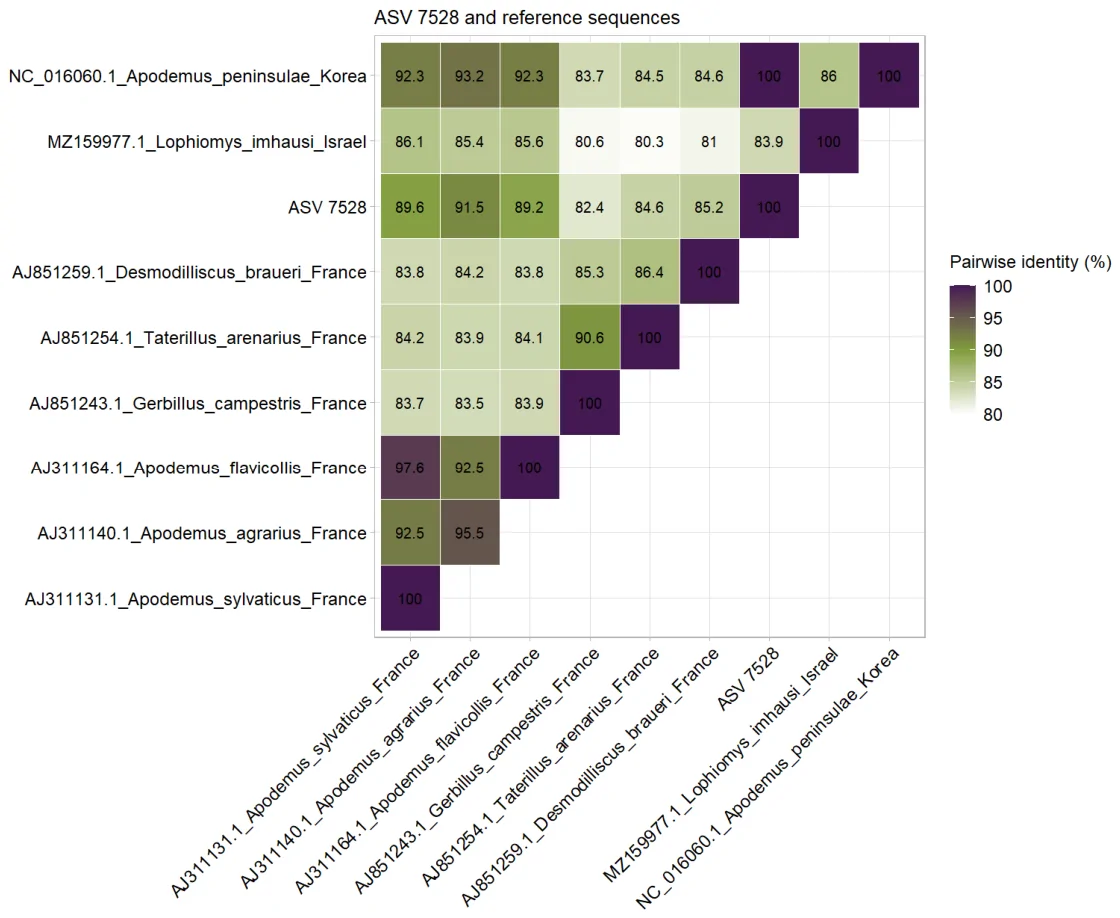
Pairwise sequence identity of ASV 7528 with reference sequences from the family Muridae. The colour key indicates the pairwise percent identity.

Phylogenetic trees were constructed for the two MiMammal-identified species to provide additional taxonomic validation and confirm their presence within the environmental samples. As of October 2026, out of six mammalian orders identified in Uljin forest, Korea, the species record coverage (NCBI database) of *COI* and MiMammal (i.e., *12S rRNA*) is 64% and 80%, respectively, illustrating a gap of 16% in record coverage between the two primers (Table S4). Representative sequences for each species, ASV 1152 for *M. leucurus* and ASV 7528 for *A. peninsulae*, were used for the phylogenetic trees. Figure 6A illustrates the phylogenetic relationships between ASV 1152 and other species belonging to Mustelidae. ASV 1152 exhibited a high phylogenetic difference from the mountain weasel (*Mustela altaica*) and the ferret-badger (*Melogale moschata*), while clustering in a single lineage with the badger sequences. Of these, ASV 1152 had the closest similarity with a *Meles leucurus* sequence reportedly found in Korea. These observations were also reflected in a pairwise identity analysis (Figure A2). The pairwise identity of ASV 1152 to the closest phylogenetic match was 99.6%, while that with other *Meles* species was around 95–98% and with other genera was 89–95%. Figure 6B illustrates the phylogenetic relationship between ASV 7528 and other animals belonging to Muridae. While differentiated from the gerbil reference sequences (i.e., *Desmodilliscus braueri*, *Gerbilus campestris*, and *Taterillus arenarius*), ASV 7528 formed a lineage with the rat/mouse sequences. Of these, a cluster formed with *A. peninsulae*, indicating its closer phylogenetic relationship with ASV 7528 relative to other *Apodemus* species. The pairwise identity of ASV 7528 with *A. peninsulae* was 100%, while that with other *Apodemus* species was 89–91% (Figure A3). These results provide evidence that the harvested high-resolution eDNA sequences were not artifacts, with (i.e., *M. leucurus*) or without (i.e., *A. peninsulae*) camera records, highlighting the benefits of ecology monitoring using eDNA metabarcoding.

## 4. Discussion

### 4.1. Post-Fire Vegetation Recovery’s Dependency on Fire Severity

Previous studies have reported that fire severity dictates the trajectory of post-fire successional vegetation. Severely affected areas usually experience long-term, high-temperature fires that burn all of the lower vegetation layers, along with the root systems of large plants, leaving dead woody trees and a thick layer of charred material that is high in nutrients [44,45]. Precipitation and snowmelt then carry these nutrients into the soil layers, increasing their bioavailability and re-fertilizing the substrate [46]. This nutrient influx facilitates the growth of fast-growing, nutrient-consuming seasonal herbaceous plants and small shrubs [45]. In contrast, in less severely burnt areas, mild fire exposure generally burns the lower vegetation, but the overstory often remains viable, with a post-fire survival rate that is approximately 50% higher than that of shrubs and herbaceous plants due to the shorter burning period and/or lower temperatures [47]. The high-nutrient layer resulting from the burnt lower vegetation re-fertilizes the soil, with the surviving large trees becoming the primary consumers of this nutrient source [44]. Therefore, seasonal herbaceous plants and shrubs need to compete with the large plants with intact, complex root systems and cannot grow as strongly as in the severely damaged sites [48]. The spatial observations in the present study were thus in line with these successional processes, and three years later, the effects of the forest fire on the landscape were still evident, as reflected by the satellite images and dNBR values.

### 4.2. Mammalian Biodiversity Shifts and the Related Factors

Natural forests are an important and complex ecosystem that is usually characterized by high taxonomic richness and diversity [49]. Uljin Forest is generally in line with this, with many orders recorded at the C sites, which represented an undisturbed habitat with a stable ecosystem. Both traditional and eDNA-based methods detected more orders at the C sites than the A and S sites, suggesting a decrease in diversity following the fire, regardless of its severity. However, when interpreting this data, it is important to consider the limitations of the survey techniques employed. The camera traps used in the present study were designed to detect all animals; thus, they may be limited for the detection and taxonomic classification of smaller animals [4]. In particular, members of the Rodentia order are frequently underrepresented in camera trap data due to their cryptic nature and the presence of ground-level obstructions (e.g., leaf litter) that obscure the field of view [50]. Therefore, although detected at both A and S sites using eDNA, Rodentia was not recorded at these sites using the camera method. On the other hand, large mammals belonging to Artiodactyla were consistently recorded by the camera traps at all sites, while the eDNA detected them only at A sites using the *COI* primer, highlighting the efficiency of the camera method for the detection of large mammals. The lack of Artiodactyla signals in the eDNA data could be because they avoid drinking from the installed water basins (i.e., no DNA deposition) or because their DNA is destroyed under the environmental conditions (e.g., UV rays from sunlight) [4].

For all monitoring methods, the mammalian diversity and richness tended to consistently decrease with increasing fire severity. Given that the assessment period for the present study was three years post-fire, this effect of the forest fire in 2022 can be considered long-term. This could be due to fire-induced changes in the vegetation layers, which affected the distribution of the mammalian community. In particular, as shown in Figure 1A–C, the S sites were characterized by a thickening of shrubs and herbaceous plants, while A sites retained an intact overstory canopy. Shrubs and herbaceous plants provide insects with food resources, shelter, and oviposition sites, which increases their abundance [51]. Furthermore, the loss of canopy cover at the S sites increases the accessibility of these enriched insect communities to Chiroptera, which are primarily insectivores [52]. Simultaneously, the growth of herbs and shrubs decreases the visibility of large herbivores (i.e., Artiodactyla) but provides them with an abundant food source [53]. In the absence of an apex predator—as evidenced by both eDNA and camera trap datasets—the enrichment of food sources at the S and A sites had a greater effect on the distribution of Artiodactyla than the danger of a loss of visibility, thus decreasing the diversity and evenness of the mammal community in the fire-damaged areas.

### 4.3. eDNA Metabarcoding Enabled the Detection of Re-Established Eukaryotic Dynamics

Inferences of prey–predator relationships were observed for three nodes: Littorinimorpha, Araneae, and Carnivora (represented by snails, spiders, and badgers, respectively). Previous studies have reported that snails are prey for some spiders and that carnivores, such as badgers, feed on both snails and spiders [54,55]. Thus, an increase in snails and spiders would attract natural predators, creating positive linkages between these orders. Although not directly related through a prey–predator relationship, the positive correlations between Artiodactyla (goats and deer) and Orthoptera (insects), and Pinales (pine trees) with Rosales (shrubs) and Lamiales (herbaceous plants) can be explained by their shared habitats. Some species belonging to the order Orthoptera thrive in open, sunlit forest patches [56]. Members of Artiodactyla are grazing mammals, feeding on the shrubs and herbaceous plants, thus clearing out the understory of the forest. Moderate grazing by Artiodactyla has been reported to benefit Orthoptera, explaining the positive linkage between these orders [57]. The positive correlations observed between Pinales and the orders Rosales and Lamiales likely originate from two key characteristics of their post-fire recovery. First, pine trees tend to sprout early and rapidly after a fire event [58], while shrubs and herbaceous plants tend to be fast-growing [59]. The positive linkages between them were thus likely due to synchronized recovery responses to the fire disturbance based on their growth characteristics. Secondly, pine trees are a vital component of Uljin Forest, modifying the soil and microclimate to support the growth of understory orders such as Rosales and Lamiales. Soil nutrients can be enriched by the accumulation of feces and food waste from animals under the plant canopies or trapped by the root systems of large woody trees, while the microclimate is mediated by the canopy to favor the growth of the lower vegetation layer by reducing exposure to frost stress during winter and intense sunlight [60,61]. Overall, the constructed Bayesian network not only reflected ecological interactions between orders but also revealed positive signs of ecosystem dynamics re-establishment, with strong signals for known ecological relationships and no association with the fire-severity index. These important insights gained from the use of eDNA with the universal *COI* primer highlight the potential of eDNA for forest health monitoring and surveillance, particularly at sites where the use of traditional methods would be difficult and prohibitively labor-intensive.

### 4.4. Reliability of the Multi-Analysis Methods in eDNA-Based Studies

The multi-primer approach has been widely implemented for eDNA analysis, especially for ecological assessments and health monitoring. In the present study, two primers were used, which produced different resolutions for taxonomic classification using the harvested eDNA. As a universal primer, *COI* can be used to detect multiple eukaryotic organisms with a high taxonomic classification accuracy at the order level [27], allowing an analysis of the complex interactions between eukaryotes within an ecosystem, providing valuable insights into their roles and ecosystem health. On the other hand, a higher resolution was achieved for mammals using the MiMammal primer, which is designed specifically to detect mammalian sequences in environmental samples [28]. To confirm the presence of authentic mammalian sequences amplified with the MiMammal primer, phylogenetic tree analysis was employed, rather than relying solely on pairwise similarity. Pairwise similarity analysis between the sample sequences and reference sequences is straightforward but overlooks the evolutionary context of the taxonomic assignment [62,63]. Consequently, relying solely on similarity analysis can lead to unidentified or wrongly identified sequences, especially for taxonomic identification at the genus or species level. Phylogenetic tree construction complements pairwise methods by calculating the evolutionary distance from the sample sequence to the closest reference sequence, thus indicating if the reference sequence is sufficient to identify and characterize the sample sequence [64]. Overall, these results demonstrate that the use of multi-primer/multi-analysis methods in eDNA-based studies is required not only to validate the output of each primer but also to derive various insights that can be combined to produce a thorough ecosystem health assessment.

### 4.5. eDNA in Forest Community Monitoring: Implications and Future Perspectives

Terrestrial mammals are a common target of ecological biomonitoring due to their large size and the relative simplicity of their monitoring methods. In addition to traditional monitoring methods such as camera traps and sound recordings, eDNA analysis has emerged for more complex and in-depth studies. The present study compared the detection of large mammals in a Korean forest using camera traps and multi-primer eDNA analysis. In general, the camera data supported the eDNA results in terms of community composition and diversity (Figure 2 and Figure 3). The *COI* primer was particularly useful for accurately determining the community composition but failed to describe the mammal community at a high taxonomic resolution (Figure 5). eDNA analysis is often limited by the lack of reference sequence diversity for the target DNA fragments and the limited variation in the targeted genes for some taxa [65]. With the aid of a more target-focused primer (i.e., MiMammal) and a more complete database of reference sequences, these limitations were resolved in the present study, with a few sequences that were identified successfully at the species level (Figure 5). Sequencing errors are also a common problem for eDNA analysis due to the low quality of the source DNA, which can result in the introduction of artificial sequences to the dataset [66]. The present study sought to overcome this issue by analyzing the sequences in an evolutionary context and constructing phylogenetic trees that reflected the close relationship between the detected sequences and a reference sequence (Figure 6). The use of the traditional camera traps also complemented the use of eDNA, which sometimes produces false negatives for truly present species due to the lack of DNA in collected samples [4,66]. In the present study, the water basins were only installed for 7 days to ensure eDNA quality, while cameras were deployed for a month; thus, it was expected that the number of detected mammal species would be higher for the camera traps, as observed in Figure 5. Collectively, these results demonstrate that future studies involving the monitoring of mammals will benefit from the application of multiple primers, particularly a universal primer and a target-specific primer, together with traditional methods for the more accurate detection and taxonomic classification of harvested eDNA.

Previous eDNA-based studies have usually focused on the detection of existing organisms but have overlooked interactions within the ecosystem. One of the few ecological interaction studies that has focused on abiotic (e.g., nutrients and turbidity) and biotic factors (fish, benthos, zooplankton, phytoplankton, and microorganisms) used structural equation modeling to interpret ecological quality [67]. Another study extended this by exploring the potential interactions among biotic factors (bacteria and macrofoulers) [17], with network analysis revealing a strong negative correlation between macrofoulers and one bacterial genus (*Pseudoalteromonas*), which was proposed to be an important player in bacteria–eukaryote interactions. These previous studies highlight the potential of network analysis for the monitoring of ecological health and the importance of interactions among biotic and abiotic factors. The present study employed Bayesian network analysis to explore probabilistic dependencies among the mammalian taxa, other eukaryotic taxa, and the fire severity index [68]. Based on previous studies, the Bayesian network was utilized to quantify a range of ecosystem responses, with a particular focus on how forest fires influence functional interactions such as food webs and shared habitat dynamics. The resulting evidence of recovering ecosystem functions was in accordance with known ecological theories and confirmed little to no long-term fire effects (Figure 4). Given the increasing use of network analysis in ecological studies [69], the present study offers important implications for the use of Bayesian networks and highlights the interpretive power of network analysis for ecosystem health monitoring. It is recommended that machine learning and explainable artificial intelligence, which can be used for more complex and in-depth analysis of organism interactions, be employed in future studies to identify important and/or particularly vulnerable taxa, thus not only diagnosing ecosystem health but also assisting in decision-making for forest restoration projects.

Spatial sampling of the current study provided important insights into the ecosystem health of a Korean forest three years after a major fire. However, the concentration and distribution of eDNA are dependent on many temporal factors, such as the mammalian breeding season, winter hibernation, and the life stages of other mammalian interacting organisms (e.g., plants) [66]. Therefore, the collection of numerous samples over both time and space is important for the comprehensive analysis of ecosystem health. By increasing the sampling time points, eDNA can be used to track changes in biodiversity over time [70] and elucidate ecosystem interactions, functions, and other trends that are seasonally dependent. However, temporal sampling is limited by its high demand for human resources, poor accessibility to study sites, and the difficulty associated with preserving the quality of the collected eDNA while avoiding contamination. Innovations focusing on overcoming these limitations are now in development, including 3D-printed samples, autonomous sampling, mobile laboratories, and advanced sequencing technologies [71]. These technologies will improve the accessibility, scale, and quality of sample collection, thus improving ecosystem health surveillance and diagnosis.

## 5. Conclusions

The present study assessed the current ecological health of Uljin Forest three years after a major fire. Comparing traditional camera traps with eDNA analysis using *COI* and MiMammal primers, it was concluded that, while camera traps were suitable for large mammals (Artiodactyla) monitoring, smaller mammals (Rodentia) were more accurately detected using eDNA. Nevertheless, the community diversity assessed using different methods resulted in the same conclusion: three years post-fire, the biodiversity of Uljin Forest remained affected, with both the diversity and richness decreasing with an increase in fire severity. The broad coverage of the *COI* universal primer enabled the analysis of the interactions between mammals and other eukaryotic organisms, reflecting ecosystem health. The plants were found to be thriving, following growth patterns affected by the early stages of the post-fire recovery process, while the inferences of prey–predator relationship or habitat–occupation interactions of other eukaryotes appeared to have re-established regardless of the fire severity. Thus, three years post-fire, although the initial effects of the fire on biodiversity and richness were still evident, the recovering eukaryotic interactions inferred positive ecological signals. Additionally, the use of MiMammal allowed the eDNA sequences of individual species to be confirmed as real sequences, highlighting the reliability of the eDNA method utilized in the current study. These findings not only provide a comprehensive overview of the terrestrial mammalian community in the context of recovery after a forest fire but also contribute to the use of eDNA in the analysis of ecosystem health via an assessment of eukaryotic interactions.

## Figures and Tables

**Table 1:** Sensing dates for the assessment of the study sites.

Condition	Fire Event	Pre-Fire	Post-Fire
Date	3 March–15 April	1 March–1 April	1 March–1 April
Year	2022	2021	2025

**Table 2:** Sentinel-2 bands [25].

Number of Bands	Name	Central Wavelength (nm)	Spatial Resolution (m)
1	Coastal aerosol	443	60
2	Blue	490	10
3	Green	560	10
4	Red	665	10
5	Red-edge 1	705	20
6	Red-edge 2	740	20
7	Red-edge 3	783	20
8	NIR	842	10
8a	NIR narrow	865	20
9	Water vapor	945	60
10	Cirrus	1375	60
11	SWIR 1	1610	20
12	SWIR 2	2190	20

## Data Availability

The raw data presented in the study are openly available in the NCBI Sequence Read Archive with the accession number PRJNA1475248.

## Supplementary Materials

The following supporting information can be downloaded at: https://www.mdpi.com/article/10.3390/biology15201827/s1. Figure S1. dNBR data mapping. Table S1. Bayesian network edge probabilities. Table S2. Bayesian network stability threshold. Table S3a. Bayesian network sensitivity. Table S3b. Bayesian network sensitivity arc strength. Table S4. Records per marker for each mammal family in Korea on NCBI database (specified for Uljin forest).

## Abbreviations

The following abbreviations are used in this manuscript:

eDNA	Environmental DNA
*COI*	mitochondrial cytochrome c oxidase subunit 1
dNBR	differenced Normalized Burn Ratio
