# Supplementary material for: Mammalian–Environmental–DNA-Derived Probabilistic Modeling Reveals Re-Established Trophic and Co-Habitation Network Structure in Fire-Affected Terrestrial Communities
Source: Biology (Basel). 2026 Oct 13;15(20):1827. doi: 10.3390/biology15201827 (PMC13701414; doi:10.3390/biology15201827)
Supplement: Supplementary file 1 [file biology-15-01827-s001.zip › biology-4612052-supplementary.pdf]

Supplementary Materials

Mammalian-environmental-DNA-derived probabilistic modelling reveals re-established trophic and co-habitation network structure in fire-affected terrestrial communities

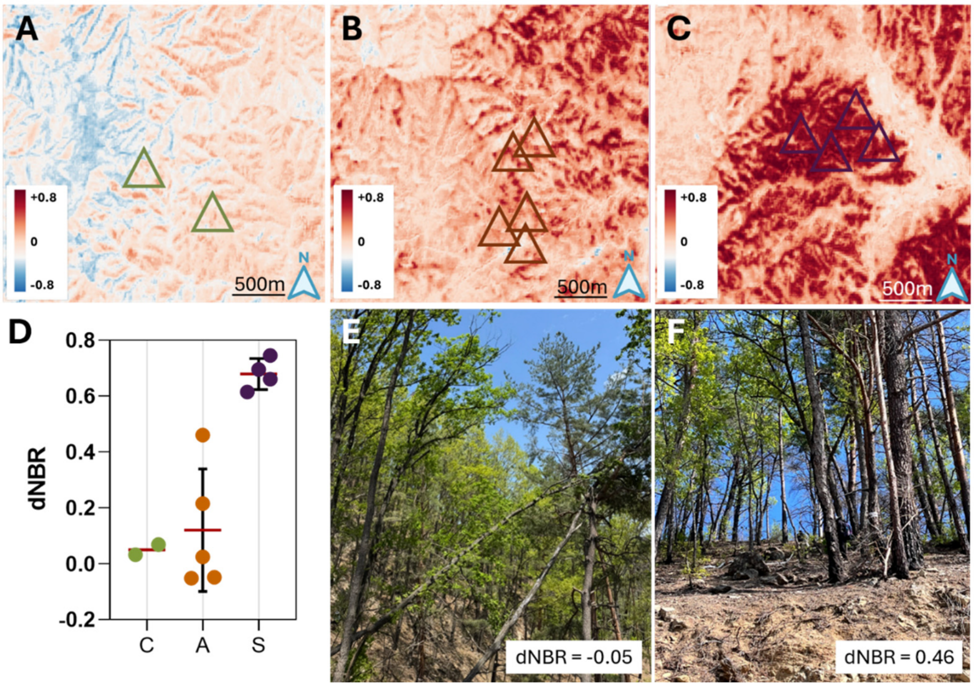

Figure S1. dNBR data mapping. The mapped dNBR over the (A) control, (B) affected, and (C) severely burnt areas between 2021 (pre-fire) and 2025 (post-fire). The locations of the sites are specified in Figure 1A. (D) Dot plot of the dNBR values at C (n = 2), A (n = 5), and S (n = 4) areas. The red lines indicate the mean. The bars and error bars represent the mean and ±1 standard deviation from the mean. The field images photographed in April 2025 show the current differences in sites and vegetation conditions of the A sites with the (E) lowest and (F) highest dNBR values recorded.

Table S1. Bayesian network edge probabilities

Arc	Strength	Direction *	Strength range runs	Runs retained	Pearson r CI	Spearman rho CI	Pearson p BH	Spearman p BH	Permutation p familywise
Artiodactyla.Goats.Deers -> Orthoptera.Insects	***0.88***	***0.596***	***0.880-0.901***	***100%***	***0.80 (-0.31, 0.97)***	***0.36 (-0.42, 0.87)***	***0.0533***	***0.818***	***0.37***
Littorinimorpha.Snails -> Araneae.Spiders	***0.807***	***1***	***0.799-0.829***	***100%***	***0.90 (-0.12, 1.00)***	***0.63 (0.00, 0.96)***	***0.0049***	***0.345***	***0.84***
Lycopodiales.Plants -> Lamiales.Plants	0.772	0.594	0.778-0.829	100%	0.53 (0.09, 0.91)	0.57 (0.03, 0.89)	0.962	0.51	0.96
Pinales.Plants -> Lamiales.Plants	***0.756***	***0.552***	***0.752-0.779***	***100%***	***0.72 (0.48, 0.92)***	***0.68 (0.50, 0.92)***	***0.196***	***0.345***	***1.00***
Symphypleona.Springtails -> Orthoptera.Insects	0.714	0.742	0.702-0.745	100%	0.13 (-0.07, 0.96)	0.44 (-0.24, 0.88)	0.962	0.737	1.00
Artiodactyla.Goats.Deers -> Chiroptera.Bats	0.689	1	0.658-0.710	100%	0.46 (0.19, 1.00)	0.67 (-0.10, 0.96)	0.962	0.345	1.00
Pinales.Plants -> Rosales.Plants	***0.688***	***0.573***	***0.652-0.707***	***100%***	***0.86 (-0.29, 1.00)***	***0.39 (-0.35, 1.00)***	***0.0163***	***0.779***	***1.00***
dNBR -> Rosales.Plants	0.677	1	0.666-0.682	100%	-0.38 (-0.75, -0.28)	-0.66 (-0.90, -0.20)	0.962	0.345	1.00
Rosales.Plants -> Lamiales.Plants	0.665	0.529	0.612-0.669	100%	0.51 (-0.23, 0.89)	0.42 (-0.21, 0.83)	0.962	0.765	1.00
Rodentia.Rats.Squirrels -> Orthoptera.Insects	0.661	0.504	0.634-0.673	10%	-0.06 (-0.27, 0.91)	0.55 (0.03, 0.84)	0.962	0.56	1.00
Rodentia.Rats.Squirrels -> Chiroptera.Bats	0.635	1	0.633-0.675	100%	-0.09 (-0.34, 0.99)	0.45 (-0.19, 0.87)	0.962	0.737	1.00
Littorinimorpha.Snails -> Rodentia.Rats.Squirrels	0.614	0.64	0.620-0.658	100%	-0.02 (-0.31, 0.96)	0.37 (-0.36, 0.80)	0.988	0.779	1.00
Littorinimorpha.Snails -> Carnivora.Felines.Badgers	***0.611***	***1***	***0.558-0.612***	***100%***	***0.96 (0.81, 1.00)***	***0.73 (0.53, 1.00)***	***< 0.001***	***0.301***	***1.00***
dNBR -> Araneae.Spiders	0.608	1	0.575-0.644	100%	-0.12 (-0.45, 0.80)	0.51 (-0.08, 0.86)	0.962	0.661	1.00
Symphypleona.Springtails -> Artiodactyla.Goats.Deers	0.604	0.679	0.587-0.628	100%	-0.16 (-0.46, 0.80)	0.16 (-0.58, 0.83)	0.962	0.927	1.00
Araneae.Spiders -> Carnivora.Felines.Badgers	***0.59***	***1***	***0.563-0.619***	***100%***	***0.97 (-0.33, 1.00)***	***0.27 (-0.41, 0.79)***	***< 0.001***	***0.927***	***1.00***
Rosales.Plants -> Carnivora.Felines.Badgers	0.58	1	0.558-0.595	100%	0.10 (-0.29, 1.00)	0.27 (-0.29, 1.00)	0.962	0.927	1.00
Littorinimorpha.Snails -> Orthoptera.Insects	0.558	0.641	0.539-0.620	100%	-0.08 (-0.36, 0.77)	0.14 (-0.54, 0.74)	0.962	0.927	1.00
Littorinimorpha.Snails -> Artiodactyla.Goats.Deers	0.556	0.608	0.539-0.588	100%	0.11 (-0.42, 0.93)	-0.20 (-0.74, 0.52)	0.962	0.927	1.00
Fagales.Plants -> Araneae.Spiders	0.552	1	0.530-0.595	100%	-0.24 (-0.61, 0.80)	-0.42 (-0.85, 0.32)	0.962	0.765	1.00
Fagales.Plants -> Lamiales.Plants	0.544	0.727	0.523-0.571	100%	0.34 (-0.14, 0.85)	0.50 (-0.17, 0.88)	0.962	0.661	1.00
Rosales.Plants -> Orthoptera.Insects	0.519	1	0.512-0.555	100%	0.13 (-0.34, 0.97)	0.07 (-0.61, 0.68)	0.962	0.936	1.00
Symphypleona.Springtails -> Rodentia.Rats.Squirrels	0.516	0.61	0.481-0.524	50%	-0.11 (-0.31, 0.67)	0.46 (-0.32, 0.83)	0.962	0.737	1.00
Rodentia.Rats.Squirrels -> Artiodactyla.Goats.Deers	0.515	0.516	0.469-0.524	30%	-0.13 (-0.37, 0.85)	0.12 (-0.48, 0.77)	0.962	0.927	1.00

Edge probabilities are conducted for 11 samples (n = 11). Confidence interval is 95% bootstrap percentile. BH was carried out for 91 pairs. Permutation p family-wise of an arc is (1 + number of permutations with a maximum strength at least as large as the arc's)/(1+50). Arcs with Pearson r values > 0.6 are highlighted in bold and italic fonts. *Direction = 1 reflects the trophic-tier blacklist, not the data.

Table S2. Bayesian network stability threshold

Threshold	N arcs
0.3	74
0.35	54
0.4	42
0.45	35
0.5	24
0.55	20
0.6	15
0.65	10
0.7	5
0.75	4
0.8	2
0.85	1
0.9	0
0.95	0

Table S3a. Bayesian network sensitivity

Scenario	N arcs	Baseline arcs recovered with the same direction	Baseline connections recovered in any direction
**Baseline**	24	1	1
**No blacklist**	24	0.38	0.58
**Score AIC-g**	27	0.88	0.92
**Hill-climbing**	19	0.75	0.79
**CLR taxa**	32	0.67	0.67

Scenarios: "Baseline" used tabu search, BIC-g score, trophic-tier blacklist, arc retained at strength > 0.5; "No blacklist" removed the blacklisting; "Score AIC-g" replaced BIC-g score with AIC-g score; "Hill-climbing" replaced tabu search with hill-climbing; "CLR taxa" applied centered log-ratio transformation for the taxon abundances (pseudocount is half the smallest non-zero value; dNBR untransformed). "N arcs" are the arcs in that scenario's averaged network. "Same direction" is the baseline acrs present with identical orientation; "any direction" ignores the orientation.

Table S3b. Bayesian network sensitivity arc strength

	Baseline	No blacklist	Score AIC-g	Hill-climbing	CLR taxa
Artiodactyla.Goats.Deers - Orthoptera.Insects	0.88	0.74	0.89	0.88	0.59
Littorinimorpha.Snails - Araneae.Spiders	0.81	0.90	0.81	0.79	0.68
Lycopodiales.Plants - Lamiales.Plants	0.77	0.77	0.79	0.78	0.79
Pinales.Plants - Lamiales.Plants	0.76	0.68	0.80	0.78	0.56
Symphypleona.Springtails - Orthoptera.Insects	0.71	0.65	0.72	0.67	0.72
Artiodactyla.Goats.Deers - Chiroptera.Bats	0.69	0.78	0.69	0.64	0.75
Pinales.Plants - Rosales.Plants	0.69	0.55	0.67	0.65	0.70
dNBR - Rosales.Plants	0.68	0.47	0.72	0.58	0.67
Rosales.Plants - Lamiales.Plants	0.67	0.44	0.65	0.61	0.42
Rodentia.Rats.Squirrels - Orthoptera.Insects	0.66	0.54	0.70	0.64	0.47
Rodentia.Rats.Squirrels - Chiroptera.Bats	0.63	0.47	0.66	0.62	0.51
Littorinimorpha.Snails - Rodentia.Rats.Squirrels	0.61	0.45	0.64	0.55	0.66
Littorinimorpha.Snails - Carnivora.Felines.Badgers	0.61	0.81	0.58	0.58	0.36
dNBR - Araneae.Spiders	0.61	0.54	0.63	0.54	0.52
Symphypleona.Springtails - Artiodactyla.Goats.Deers	0.60	0.49	0.63	0.54	0.68
Araneae.Spiders - Carnivora.Felines.Badgers	0.59	0.67	0.59	0.58	0.52
Rosales.Plants - Carnivora.Felines.Badgers	0.58	0.55	0.57	0.56	0.25
Littorinimorpha.Snails - Orthoptera.Insects	0.56	0.29	0.58	0.48	0.76
Littorinimorpha.Snails - Artiodactyla.Goats.Deers	0.56	0.33	0.58	0.51	0.72
Fagales.Plants - Araneae.Spiders	0.55	0.43	0.56	0.48	0.43
Fagales.Plants - Lamiales.Plants	0.54	0.55	0.55	0.54	0.55
Rosales.Plants - Orthoptera.Insects	0.52	0.52	0.54	0.48	0.32
Symphypleona.Springtails - Rodentia.Rats.Squirrels	0.52	0.32	0.50	0.41	0.48
Rodentia.Rats.Squirrels - Artiodactyla.Goats.Deers	0.51	0.40	0.51	0.45	0.50

Table S4. Records per marker for each mammal family in Korea on NCBI database (specified for Uljin forest)

**Order***	**Family***	**Species in Korea***	**COI Species with records in Korea**	**COI % of species with records in Korea**	**12S Species with records in Korea**	**12S % of species with records in Korea**
Rodentia	Muridae	7	5	71.43	6	85.71
Rodentia	Sciuridae	3	3	100.00	3	100.00
Carnivora	Mustelidae	9	4	44.44	5	55.56
Artiodactyla	Cervidae	4	2	50.00	4	100.00
Artiodactyla	Bovidae	1	1	100.00	1	100.00
Artiodactyla	Suidae	1	1	100.00	1	100.00
**Summary**		**25**	**16**	**64**	**20**	**80**

*Jo, Y.-S., J.T. Baccus and J.L. Koprowski, Mammals of Korea. 2018: National Institute of Biological Resources.
